# French translation, cultural adaptation and validation of the BDDQ-AS for rhinoplasty patients

**DOI:** 10.1186/s40463-019-0343-x

**Published:** 2019-05-15

**Authors:** Daniel Milad, Marie-Renée Atallah, Youcef-Hamza Benamer, Mikhail Saltychev, Sam P. Most, Sami P. Moubayed

**Affiliations:** 1 Division of Otolaryngology-Head and Neck Surgery, Department of Surgery, Université de Montréal, 5400 Boul Gouin O, Montréal, QC H4J 1C5 Canada; 2 Department of Physical and Rehabilitation Medicine, Turku University Hospital and University of Turku, P.B. 28, FIN-20701 Turku, Finland; 3 Division of Facial Plastic and Reconstructive Surgery, Department of Otolaryngology-Head & Neck Surgery, Stanford University School of Medicine, 801 Welch Road Palo Alto, Stanford, CA 94304 USA

**Keywords:** Rhinoplasty, Body dysmorphic disorder, Screening, French

## Abstract

The Body Dysmorphic Disorder Questionnaire-Aesthetic Surgery (BDDQ-AS) is a validated questionnaire that is used as a screening tool for body dysmorphic disorder (BDD) in aesthetic rhinoplasty patients. The BDDQ-AS questionnaire was translated from English to French according to international guidelines. Ten French-speaking rhinoplasty patients were interviewed in order to evaluate the understandability and acceptability of the translation and produce a final version. It was then administered to 165 consecutive patients. Psychometric properties were evaluated using item-reponse theory (IRT). Internal consistency was high, with Cronbach’s alpha of 0.90 (95% lower CL 0.88). While the discrimination abilities of all the items were good (over 2.0), their difficulty parameters were shifted towards greater severity of symptoms. That shift could also be observed in information function graph for the entire scale. In other words, the BDDQ-AS performed better in patients with more severe body dysmorphic symptoms. In conclusion, the BDDQ-AS was translated, adapted, and psychometrically validated for use in a French-speaking population.

## Introduction

The Body Dysmorphic Disorder Questionnaire-Aesthetic Surgery (BDDQ-AS) is a validated seven-item short questionnaire used to screen for body dysmorphic disorder (BDD) [1]. Screening is positive when the patient is concerned (question 1 = yes) and preoccupied with their appearance (question 2 = yes) and that these concerns caused at least moderate distress or impairment in daily life (question 3, 4, 5 or 6 equal or greater than 3, or question 7 = yes) [1]. It has been shown that rhinoplasty patients who screen positive on the BDDQ-AS were significantly less satisfied with the final results of surgery [1]. There is currently no available translation of the BDDQ-AS questionnaire in French. We aim to carry out the translation, cultural adaptation, and validation of the BDDQ-AS questionnaire for French-speaking rhinoplasty patients.

## Methods

Translation and cultural adaptation was conducted according to international guidelines guidelines [2]. Standard forward and back-translation procedure was followed. Two independent certified translators produced two forward translations that were merged. A third translator back-translated the reconciled version for review and identification of discrepancies. Interviews were then conducted with ten rhinoplasty patients that were native French speakers to identify ambiguities and to verify understandability and acceptability. For validation, one hundred and sixty-five consecutive rhinoplasty patients completed the final questionnaire. Internal consistency was defined by a Cronbach’s alpha reported along with its one-sided (lower) 95% confidence limit (95% CL). To investigate the correlations between the items included in the BDDQ-AS scale, the Spearman correlation coefficient was obtained along with a two-tailed *p*-value.

Item response theory (IRT) analysis defines discrimination and difficulty variables of a questionnaire. A discrimination variable describes the sensitivity of the test to differentiate different severities of symptoms [3]. In turn, the difficulty variable refers to the level of a perceived nasal problem needed to achieve a 50% probability of choosing a particular score [3]. The IRT Rating Scale Model was used. Item information and test characteristic curves and the test information functions were presented graphically.

All analyses were performed using Stata/IC Statistical Software: Release 15, College Station (StataCorp LP, TX, USA). All *p*-values were considered statistically significant if = < 0.05 (when not mentioned otherwise).

## Results

**Fig. 1:**
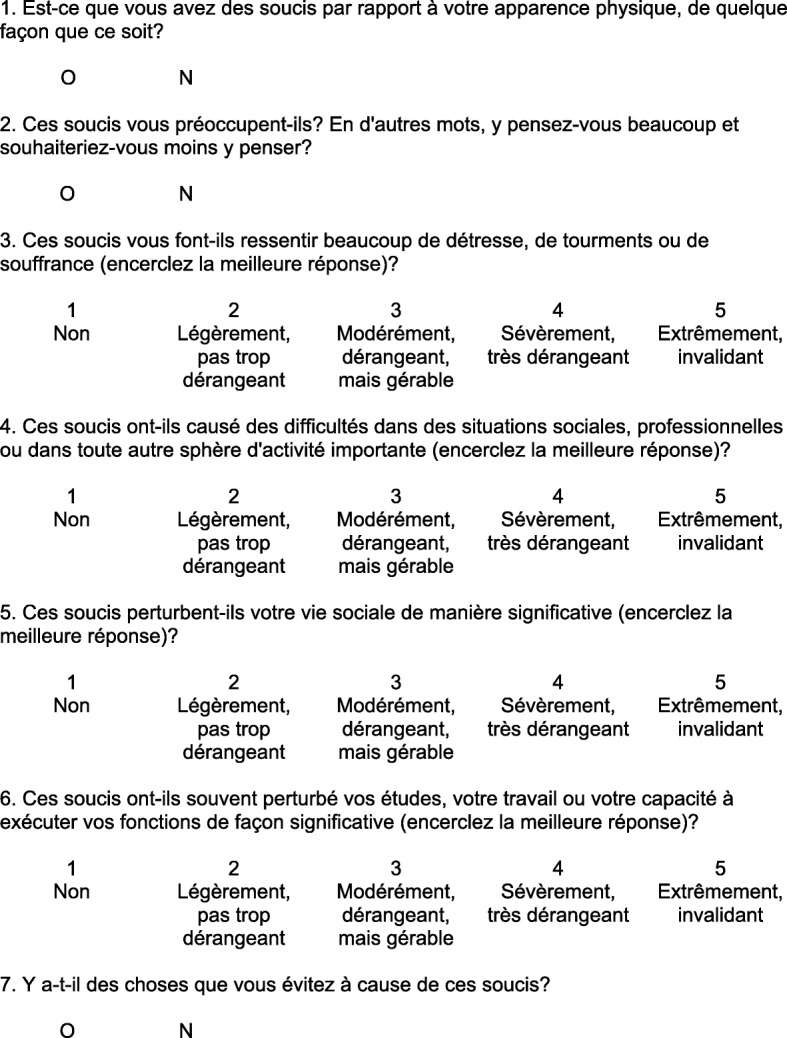
French BDDQ-AS (F-BDDQ-AS)

There were a few differences between the two forward translations, which were reconciled and harmonized. Back-translation showed only minimal discrepancies with the original concepts requiring no further modification. Ten interviews were conducted with two preoperative and eight postoperative rhinoplasty patients (four women, six men, mean age 39.4). No modifications were made after cognitive interviews. The final version of the French version is seen in Fig. 1.

**Fig. 2:**
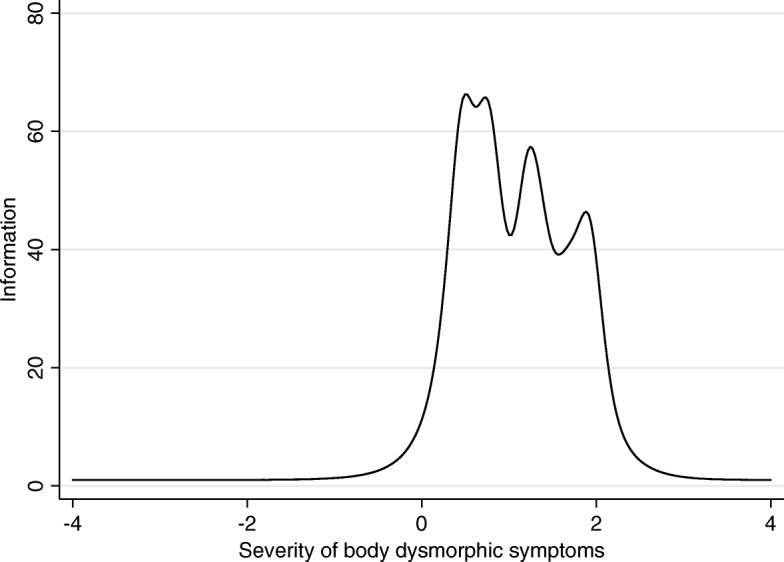
Test information function for BDDQ-AS

The results from IRT analysis are available from 165 individual patients (75 females, 89 males, 69 septorhinoplasty, 95 non-septorhinoplasty; mean age 48.6 years), shown in Table 1. Based on the BDDQ-AS questionnaire, 40 patients had body dismorphic disorder (40%). The internal consistency of BDDQ-AS was good with Cronbach’s alpha 0.90 (lower 95% CL 0.88). While the discrimination abilities of all the items were good (over 2.0), their difficulty parameters were shifted towards greater severity of symptoms (Table 1). That shift could also be observed in information function graph for the entire scale (Fig. 2).

**Table 1:** Discrimination and difficulty abilities of BDDQ-AS items

	Estimate	95% CI	
BDDQ-AS 1			
Discrimination	2.52	1.51	3.54
Difficulty	0.43	0.22	0.64
BDDQ-AS 2			
Discrimination	3.47	2.02	4.92
Difficulty	0.48	0.29	0.66
BDDQ-AS 3			
Discrimination	5.31	3.50	7.12
Difficulty			
> =2	0.31	0.16	0.47
> =3	0.63	0.46	0.80
> =4	1.33	1.07	1.58
5	1.84	1.46	2.23
BDDQ-AS 4			
Discrimination	10.38	4.82	15.93
Difficulty			
> =2	0.49	0.34	0.63
> =3	0.74	0.58	0.90
> =4	1.21	0.99	1.44
5	1.93	1.54	2.32
BDDQ-AS 5			
Discrimination	8.69	5.14	12.24
Difficulty			
> =2	0.44	0.29	0.59
> =3	0.81	0.64	0.98
> =4	1.36	1.11	1.61
5	1.65	1.33	1.97
BDDQ-AS 6			
Discrimination	3.80	2.41	5.20
Difficulty			
> =2	0.77	0.57	0.96
> =3	1.02	0.79	1.24
> =4	1.53	1.19	1.86
5	2.08	1.55	2.61
BDDQ-AS 7			
Discrimination	2.61	1.47	3.75
Difficulty	0.83	0.59	1.08

Hybrid models with 2-parameter logistic sub-models for BDDQ-AS 1, BDDQ-AS 2, and BDDQ-AS 7 and graded response sub-models (RSM) for BDDQ-AS 3, BDDQ-AS 4, BDDQ-AS 5, and BDDQ-AS 6 items. The *p*-values for all the estimates < 0.001

## Discussion

We were able to translate, adapt, and validate the BDDQ-AS into French, producing the F-BDDQ-AS. This French version was shown to be conceptually and psychometrically equivalent to the original English version. The meticulous process of translation and cultural adaptation is supported by many international guidelines [2, 4, 5].

The F-BDDQ-AS is a reliable instrument, as demonstrated by a high internal consistency. These results are very similar to the Cronbach’s alpha of the original English version [1]. It is also a valid instrument, which refers to its ability to measure accurately the outcome of interest. Multiple analyses are required in order to prove validity [6], as it has already been demonstrated for the original English version of the BDDQ-AS [1]. The positive and significant correlation between each item of the F-BDDQ-AS is a demonstration. The methodology used for the translation process is also a safeguard of content validity.

The small number of participants recruited for the psychometric validation is a limitation to this study. However, many similar translation studies achieved a validation process with similar or a more limited number of participants [7–9]. Furthermore, a small sample size would only diminish the chances of finding a significant association. Since our results showed the reliability and validity of the French BDDQ-AS, especially with IRT,we believe the associations would only be stronger with a larger sample size. In other words, the BDDQ-AS performed better in patients with more severe body dysmorphic symptoms.

This study is first to generate a French version of the BDDQ-AS questionnaire. Adapted questionnaires are important in health-related quality of life evaluation. They are useful for screening and monitoring the individual patient, for the evaluation of health outcomes (1467690), and for providing comparable results for international research [5].

## Conclusions

In conclusion, we successfully translated, adapted, and validated the BDDQ-AS into French in order to help with the evaluation of functional and cosmetic outcomes of rhinoplasty patients. We hope this will provide an \itional tool to clinicians who are evaluating French-speaking rhinoplasty patients.
